# A Review of Patient Experiences and Provider Education to Improve Transgender Health Inequities in the USA

**DOI:** 10.3390/ijerph20206949

**Published:** 2023-10-20

**Authors:** Gabriel J. Tanenbaum, LaTasha R. Holden

**Affiliations:** 1 Department of Psychology, University of Illinois Urbana-Champaign, Champaign, IL 61820, USA; lrholden@illinois.edu; 2 Beckman Institute, University of Illinois Urbana-Champaign, Urbana, IL 61801, USA

**Keywords:** attitudes toward transgender people, transgender healthcare inequities, healthcare provider education, educational interventions, access to care

## Abstract

Transgender individuals are an underserved, vulnerable population. They face many inequities including barriers in both accessing and receiving adequate healthcare. These inequities are proposed here to be rooted in a lack of education about transgender people and their experiences. We begin by exploring the existing transgender healthcare research carried out in the USA, examining client experiences, provider education and attitudes, and the barriers transgender people face to obtaining proper healthcare. Secondly, we look at the previous research on educational interventions implemented with medical students and practitioners in the USA to enhance knowledge about transgender people, and increase sensitivity and awareness, while also increasing the level of comfort in working with these clients. The limitations in these fields of study are discussed in order to understand how to better serve transgender clients in the USA. We will do this through a narrative review to determine evidence-based best practices for educational intervention, uncovering gaps in the literature and highlighting where to focus in future work for researchers and practitioners.

## 1. Introduction

The past few years have seen a sharp rise in legislation targeting transgender people in the USA. Since the beginning of 2023, 49 out of the 50 states have introduced anti-transgender bills, with a countrywide total of 567 bills [1]. Compared to 2022, with 155 bills [2], this year, so far, we have seen the number of bills triple. Recent data from the Pew Center demonstrated that 64% of Americans favor laws that would protect transgender people from discrimination, whereas the percentage who believe that a person’s gender can be different from the sex assigned at birth was only 38% in May 2022, a decrease from 44% in September 2017 [3]. Using the definition of transgender as “a person whose gender identity or expression is different from their sex assigned at birth” [4], there has been a decline in the belief that transgender people’s existence is valid [3]. Trends in the legislation and beliefs about transgender people in the USA are worrying, given the vulnerability of the transgender population. This vulnerability includes a variety of psychosocial issues, such as exposure to violence, discrimination, financial difficulties, drug and alcohol misuse, problems accessing health and social care services [5,6,7], and an increased risk for contracting sexually transmitted infections and human immunodeficiency virus [8,9].

Transgender people in the USA have also been found to experience a high level of stigma and discrimination which limit their opportunities and access to resources in critical domains including employment and healthcare [4]. A large-scale study on transgender people in the USA found that they had worse socioeconomic outcomes (compared to their cisgender counterparts), including being significantly less likely to have a college education, and having significantly lower employment rates and a lower household income [10]. Transgender people in the USA have also been found to have a lower prevalence of health insurance coverage compared to the general population [11]. These and other factors all play a role in transgender people’s quality of life and their ability to access healthcare. 

Transgender people are also subject to minority stress, which can be defined as “the psychological and physiological consequences associated with having a marginalized identity” [12]. Transgender people of color and indigenous people experience further stress due to having multiple marginalized identities, conceptualized as multiple minority stress [12]; for further reading, see [13,14]. These stressors play a role in many aspects of their lives, including accessing physical healthcare, which is the present focus. In a statewide assessment of transgender Virginians, 41% reported experiencing trans-related discrimination in healthcare [15] and, in the 2010 National Transgender Discrimination Survey, 28% of participants reported postponing medical care due to discrimination [16]. 

### 1.1. Other Healthcare Barriers for Transgender People

Discrimination is not the only barrier for transgender people seeking healthcare, with 48% reporting the postponement of medical care due to the inability to afford it, and 19% reporting being refused care due to their status as transgender/gender-non-conforming (TGNC) [16]. The inaccessibility of gender-affirming care can have dire consequences, as it has been reported that the suicide rate is greater for transgender and gender-diverse individuals when they desire gender-affirming care but find it inaccessible [17]. Transgender individuals have also been found to access both primary and specialty care services at a lower frequency than their cisgender counterparts [18]. Access itself is an issue, with 23.6% of transgender Massachusetts residents reporting being unable to access transition-related care [19]. These statistics are all concerning and highlight the difficulties that transgender people face in accessing and receiving adequate healthcare. 

### 1.2. Healthcare Concerns for Transgender People

When surveyed on their health and healthcare concerns, TGNC individuals’ top three concerns were found to be insurance coverage for transition-related care, access to, and the availability of, transition-related care, and the education of healthcare providers (HCPs) about transgender patients and issues [20]. This concern about the education of HCPs regarding transgender people has been supported through several studies finding that HCPs felt uneducated about treating transgender patients [21,22,23]. Another study found that the biggest barrier for healthcare reported by transgender individuals was a lack of access due to a lack of knowledgeable providers [24]. Issues with HCPs are a prominent problem for transgender people accessing care and have been supported by further literature as well [25,26]. The lack of education among professionals is a persistent problem, with transgender people having reported being required to teach mental health practitioners about their issues and barriers to care (see [27,28,29]). Transgender people are certainly at a disadvantage when seeking care, and reform is needed in order to improve their experiences.

### 1.3. Methodology

This paper will begin by reviewing the literature on transgender patient experiences in order to provide a greater insight into the specific discrimination and barriers that TGNC individuals face when seeking healthcare. This will take the form of a narrative review, which was chosen to allow for more flexibility in the inclusion of articles [30,31]. The papers included in both parts of the review were primarily found through Google Scholar and through snowballing, where relevant papers’ reference lists were searched for additional relevant references. There was no restriction on the date range of papers, though all of the research has been conducted in the past two decades. In the first part of the review, the papers were found using the search term, “transgender healthcare experiences”. For the second part of the review, which focuses on HCPs’ education around transgender people, these papers were found using the search terms of “transgender health education”. The selection criteria for these papers were that they involved physical HCPs and took place in the USA in order to keep a concise scope. This narrative review aims to provide researchers and practitioners with a baseline understanding of both transgender healthcare experiences and the previous interventions conducted with HCPs. 

This review is needed based on the aforementioned findings on the negative experiences with providers faced by transgender people [15,16,20], which warrants a deeper look into the attitudes toward transgender people and potential biases against transgender people among HCPs. We will also review the educational intervention literature on this topic to allow for a summary of the work already carried out and to also make recommendations for future research to improve transgender attitudes and client experiences with HCPs. An important note for this work is that it is non-exhaustive and focuses solely on the USA. We aim to provide a comprehensive look at transgender experiences in the healthcare system in the USA, as well as the attitudes and education of providers in this context. This focus was chosen due to the aforementioned challenges that have arisen against transgender rights in the USA in recent years. Further, this work centers on transgender adults for sake of scope.

## 2. Patient Experiences with Healthcare Access and Providers

The research on the experiences of transgender patients with HCPs has been relatively little, but recent efforts have been undertaken in order to better study this topic. Two notable large-scale national studies have been conducted in the USA, the 2015 United States Transgender Survey [32] and the 2010 National Transgender Discrimination Survey [16]. The data from these two studies have been analyzed for a variety of outcomes, which will now be detailed before the review of smaller-scale studies.

### 2.1. The 2015 United States Transgender Survey

The 2015 United States Transgender Survey is one of the most prominent studies on transgender healthcare experiences in the USA thus far [32], and collected data from 27,715 adults who identified as transgender. These data have been analyzed in numerous studies to examine different aspects of healthcare access for transgender individuals. From the survey, one in three participants reported having at least one negative experience in healthcare related to gender identity in the past year [33]; this was found along with both interpersonal (e.g., a lack of provider sensitivity or knowledge, the refusal of care for transgender patients, and harassment) and anticipation barriers (i.e., the avoidance of healthcare due to the anticipation of mistreatment or discrimination from providers) to accessing healthcare. Further, when actively seeking healthcare, eight percent of participants reported denial of trans-specific healthcare, and around three percent reported refusal of general healthcare [34]. These negative experiences were more likely to occur for transgender clients of an older age, biracial- or multi-racial-identified individuals, people with disabilities, and lower-income, and less-educated individuals [34]. Transgender individuals with another marginalized identity are at a higher risk for healthcare discrimination, receiving lower-quality care and receiving care from providers with limited competence [34]. Further, these findings were replicated in additional works by Romanelli & Lindsey 2020 [35] and Seelman et al., 2021 [36]. 

In terms of healthcare utilization, the strongest predictors of visiting an HCP were found to be the perception of HCP knowledge, health insurance status, and the costs of healthcare [37]. For those who did utilize healthcare, the experience of stressors (e.g., the refusal of care, inadequate provider knowledge, or invasive irrelevant questions about transgender status from providers) was found to be associated with symptoms of emotional distress and greater odds of physical impairment [38]. Another recent study that used data from the 2015 U.S. Transgender Survey [32] along with data from the PRIDE Study [39] found that 70.1% of transgender, nonbinary, and gender-expansive people who were assigned female or intersex at birth reported at least one negative HCP interaction in the past year, with those who were specifically seeking gender affirming care being 8.1 times more likely to report a negative interaction [40]. This large-scale study has provided a valuable source of information on transgender healthcare experiences. Likewise, these findings shed light on the specific challenges that transgender clients face, which could lead to efforts to remedy these issues, including improving provider education. 

### 2.2. The 2010 National Transgender Discrimination Survey

Another large-scale study on transgender people in the USA was conducted in 2010, the National Transgender Discrimination Survey (NTDS) [16]. In this survey, it was found that 30.8% of participants reported delaying or not seeking healthcare, with delays in care being four times more likely among those who reported having to teach HCPs about transgender people [41]. The refusal by HCPs to provide care was found to be positively associated with individual-level client factors, including older age, a trans-feminine identity, a lower income, and identification as multi-racial or of another ethnic/racial minority [42]. Other analyses have found that transgender people of color experience higher levels of anti-transgender discrimination compared to their White counterparts [43]. The NTDS data were also compared to National Health Interview Survey data, and it was found that TGNC were less likely to have health insurance and a primary care physician compared to cisgender people [44]. Collectively, these findings from the NTDS highlight the severity of discrimination faced by transgender people in the USA and the need for HCPs to work to better serve them.

### 2.3. Other Small-Scale Transgender Healthcare Studies

Aside from these two large-scale studies, additional smaller-scale studies on transgender healthcare experiences in the USA have been conducted, as well. One such study was the 2014 Colorado Transgender Health Survey, which included about 400 participants, of whom about 40% reported delaying healthcare [45]; those who did were also found to have worse general health [46]. Additionally, one of the strongest indicators for not delaying healthcare was having access to a trans-inclusive primary care provider [47]. These data further suggest that fear of discrimination and the consequential delay of care are potentially large healthcare challenges for transgender adults. 

More evidence of barriers to care has been found using the 2018 Pride Study data. Clark et al. 2022 found that transgender people were more likely to avoid healthcare when having reported previous mistreatment. This relationship was further examined using logistic regression models, with interaction terms added to evaluate whether state-level healthcare protection policies moderated the relationship between healthcare mistreatment and healthcare avoidance due to anticipated mistreatment [48]. They found no significant moderating relationship. This lack of improvement in transgender healthcare experiences, despite state-level laws intended to protect them, calls for a need to critically examine the enforcement of state-level healthcare policies to ensure proper implementation [48]. 

A study conducted on 18-to-26-year-old transgender people in the USA found that they were less likely to have received a routine check-up compared to the general population [49], though it should be noted that the sample size was very small, only including 34 participants. Another small-scale study involved a focus group in Virginia that found barriers to accessing healthcare, such as poor patient–provider relationships, a lack of insurance related to employment discrimination, and issues with identity documentation [50]. Many of these participants also mentioned hostility, insensitivity, disrespect, and discrimination in their experiences with HCPs. 

Looking more specifically at direct experiences with HCPs, studies have found issues of gatekeeping (i.e., using power to withhold care), pathologization (i.e., attributing transgender identities to mental illness), and stigmatization (i.e., labeling and discrimination against marginalized individuals) [51]. Additional issues when accessing healthcare included misgendering by HCPs, unfamiliarity with transgender people and their health issues, and transphobic practices (e.g., pathologizing gender difference, discomfort with transgender people, and denial of care to transgender people) [52]. Other issues have been found, including the high costs of medical care and a lack of transgender-friendly providers, to be barriers to care [53]. An online study of US transgender adults found that mistreatment by providers could be categorized according to six overarching themes: gender insensitivity, displays of discomfort, denied services, substandard care, verbal abuse, and forced care, which consisted of involuntary psychiatric commitment and unnecessary examinations after transgender status disclosure [54]. 

Though there have been many issues identified in transgender healthcare in the USA, there have also been some positive findings. For patients who had a provider with some level of knowledge about transgender people, this was found to be one of the most critical factors for increasing healthcare utilization [37]. Transgender people who were under a physician’s care were also found to be more likely to obtain hormone therapies from a licensed physician and to have reduced high-risk behaviors [53]. The use of inclusive language and demonstrated experience with transgender patients were two other factors found to lead to positive experiences, including providers’ use of language that respects gender diversity, demonstrated knowledge of, and experience with, transgender health, and the treatment of identity disclosure as routine [52]. Another finding was that transgender patients had better experiences with nurses in LGBTQ or transgender-specific health centers [55]. This emphasizes the need for inclusive support from HCPs to increase their education on transgender people. Together, all of this evidence from transgender experiences emphasizes the need for provider education and other improvements to aid healthcare access for transgender people. Next, the other side of this issue will be examined, through a review of previous research on HCP attitudes and education on transgender people in the USA in order to identify the gaps in, and limitations of, previous research and suggest improvements for future research and practice. 

## 3. Provider Attitudes and Education

As previous research has highlighted the importance of providers in access to healthcare for transgender people, it is vital to study and work to improve providers’ attitudes and education regarding transgender people. There have been several studies that have investigated one or both of these topics. In the large-scale 2015 U.S. Transgender Survey, 17.8% of participants reported that their primary care provider knew almost nothing about transgender health [56]. Likewise, evidence has been found that providers feel uneducated and uncomfortable when it comes to treating transgender patients, including in a survey of internal medicine residents [21]; newly graduated medical students [22]; and primary care providers in an integrated healthcare system [23]. In the survey of internal medicine residents, only 45% reported any prior education about transgender care, while less than one third of them felt comfortable prescribing gender-affirming care or referring patients to another physician to receive such care [21]. The study on primary care providers in an integrated healthcare system found that almost half did not receive formal education on transgender patients (48.4%), and yet 49.7% of them reported having worked with a transgender patient before [23]. This illustrates that the level of medical education garnered does not help in meeting the needs of current transgender clients. The regression analysis performed from the aforementioned study [23] found a negative association between provider knowledge of transgender health and transphobia, but no significant association was found between hours of formal/informal education and transphobia; this suggests that education may not be enough to improve the biased beliefs held by medical professionals and highlights the need to address transphobic beliefs in addition to educating providers.

Regarding providers, a more positive finding was observed when surveying health professional students, with 67% reporting a high level of personal comfort in caring for a transgender patient [57]. However, all of these students reported only low or intermediate levels of knowledge and skills in terms of caring for these patients. This suggests that healthcare students are willing to care for transgender patients but need more education in order to do so effectively. In a survey of medical school deans, the median reported time dedicated to teaching LGBT-related content in the entire medical school curriculum was only five hours [58], which means even less of that short amount of time was spent dedicated to transgender health, as they are a subset of the LGBT population. 

In a survey of US academic medical practices, only 16% reported comprehensive LGBT training, while more than half (52%) reported providing no LGBT training at all [59]. Further work looking at medical students’ education and attitudes regarding LGBT patients found a median of 22 h of curricular time devoted to LGBT health, and that 93% of the students had cared for five transgender patients or less during their time in medical school, and 40% had not cared for a single one [22]. However, there was a significant positive association between patient exposure and comfort providing care, as well as between curricular hours and comfort providing care. This points to a need not only for increased education around caring for transgender patients but also a need to increase exposure to transgender patients in order for practitioners to gain comfort and familiarity with them.

A qualitative study conducted among clinicians in the US regarding transgender healthcare looked at how they obtain and use information, perceive barriers, and understand gaps in their own professional training [60]. The clinicians reported increased comfort and competence when they underwent mentorship, self-directed learning, clinical experience, and person-centered harm-reduction approaches. The data for mental health care professionals have been found to be slightly better than those for physical HCPs, with 81% reporting in a survey that they received specific training about working with gender-diverse clients [61]. Those mental health care professionals also reported high levels of comfort, competence, and ability to work with these clients, which were statistically significantly associated with the number of hours of transgender and gender-diverse training they had received [61]. Of these participants, only one third reported that gender diversity was part of their education in graduate mental health training [61].

Though previous research has found that current early-career clinicians have more formal schooling on transgender health than in the past, these data suggest that formal education is not a cure-all for improving transgender healthcare. Not only that, but contemporary data support the belief that gaps still exist in the curriculum [62,63,64,65], and biases against transgender people may continue in spite of education [23]. It should be noted that the biases held by individuals may be difficult to change due to deep-seated beliefs; however, education and exposure to transgender clients can be a good start. Combatting forms of bias requires nuance, and a combination of approaches may be needed to do so. This has all provided a picture of the current state of HCP education and attitudes toward transgender patients, which leads to the question: how can these be improved?

## 4. Intervention Research Conducted So Far

There is clearly a need for work to improve providers’ education and attitudes toward transgender patients. A promising finding from a structured literature review was that all the published educational interventions on medical students and residents proved effective for improving the attitudes around, understanding of, and/or the skills needed to be clinically competent with, transgender patients [66]. We will now review the interventions that have been performed with medical students and residents in the USA, of which there has been a greater quantity. Following this, we will review the interventions conducted with existing providers, which have not been as numerous. 

### 4.1. Intervention Performed with Medical Students and Residents

There has been much interventional work carried out with healthcare students, involving different methods, including workshops [67,68], discussion-based seminars [69], elective classes on LGBTQ health [70,71], simulation activities [72,73,74], and curriculum additions focusing on LGBT or transgender health [75,76,77,78,79,80,81], as well as online training programs [82,83,84] or structured cooperative learning exercises [85]. It is important to note that, out of these 19 studies, only two [83,84] employed a control condition, and only one of these [84] also employed randomization. The majority of the remaining studies [68,69,71,75,76,77,78,79,80,82,85] employed a pre- and post-test design, where knowledge and/or attitudes were measured before and after the intervention. Several other studies only measured post-intervention impacts on knowledge and/or attitudes toward transgender people [72,73,74], and the remaining three studies reviewed used more informal designs without surveying participants following the intervention [67,70,81].

#### 4.1.1. Workshops

According to the research, workshops that were collaborative and incorporated input from transgender individuals, HCPs, and public health researchers were found to improve the knowledge and attitudes of the healthcare students in an immediate post-intervention survey [67]. Another workshop that was designed to enhance residents’ empathy for, and treatment of, transgender patients was shown to increase immediate post-intervention empathy, knowledge, comfort, and motivations for future learning, but these significant improvements were no longer seen at a 90-day follow-up [68]. While the immediate post-intervention results of these workshops are promising, the lack of lasting effects in the latter study calls into question the true effectiveness of single-instance workshop interventions. Another related intervention involved a single 90 min discussion-based seminar with third-year medical students and included different activities including didactic teaching, multiple-choice questions, clinic vignettes, and role-playing that involved scenarios in LGBTQ clinics. These were found to produce post-seminar improvements in students’ comfort with LGBTQ patients, self-efficacy, and knowledge [69]. 

#### 4.1.2. Elective Courses

Other efforts to improve healthcare students’ knowledge around transgender patients have included elective courses or lectures offered to medical school students and residents. One elective was on LGBTQ health broadly and showed promising initial results, including medical students feeling prepared to work with LGBTQ patients and expressing satisfaction with the curriculum [70]. The curriculum used there has also been made into a publicly available toolkit by the University of Louisville School of Medicine, so that it can be used by other educators [86]. This can serve as a good example for other curriculum developers.

Another elective curriculum addition was offered to medical residents in the form of a one-hour lecture on hormone therapy for transgender patients; this was found to significantly increase the reported willingness to help with hormone therapy and increase the belief that transgender patients should be offered gender-affirming care [80]. This was a good first step toward improving residents’ knowledge and comfort working with transgender patients. Later, the same medical school piloted a transgender medicine elective that allowed students to provide clinical care to transgender individuals and found significant improvements in both comfort with, and knowledge of, transgender people [71]. This exemplifies that, while incorporating transgender specific content into medical school curricula is useful, exposure to transgender patients in a clinical setting during HCPs’ clinical years can further help narrow the knowledge and comfort gap to improve access to care for transgender people.

#### 4.1.3. Curricular Additions

In addition to elective courses, a few programs have gone a step further and created non-elective curricular additions on transgender health topics. Other work was conducted by a college of medicine that implemented two such additions; one was part of a Social Determinants of Health Orientation Program (SDHOP) that was given to first-year medical students and involved discussion groups [75]; the other was a one-hour lecture given to third-year medical students [75]. Both led to positive results, with increased comfort, competence, and/or knowledge reported by students [75,76].

Another school of medicine added required curriculum content, with one approach focusing on the “biologic evidence of durability of gender identity” in the first-year medical student curriculum [77] and another that added transgender medicine content to the second-year endocrinology unit [78]. The second-year medical students reported a 67% reduction in discomfort toward transgender patient care [78], while the first-year students showed significant increases in correct answers when tested on transgender health knowledge [79]. Enhanced knowledge and comfort are both important parts of working to improve HCPs’ treatment of transgender patients, and integrating both interventions [78,79] could be a way to maximize benefits. 

Attempts have also been made to develop and incorporate transgender health content into baccalaureate nursing education and have been found to be well received by students and faculty [81]. Another medical school also developed and implemented a curriculum around transgender health, which was given to second-year medical students and consisted of five online modules, a quiz, a three-hour case-based workshop, and a two-hour interactive patient–provider panel [87]. The post-assessment results showed statistically significant improvements in competency with gender identity and self-reported knowledge of transgender health, but the attitudes and interest in transgender issues outside of healthcare did not change significantly [87]. 

A different medical school developed a curriculum on LGBTQ health which took a cultural humility approach that included a one-hour lecture followed by a one-hour panel with LGBTQ-identified individuals and readings given before the session [77]. In the post-survey results, participants reported more positive attitudes and better scores on knowledge questions. The combination of a lecture and panel is an innovative approach that could provide the benefits previously seen from both approaches and should be further investigated. 

#### 4.1.4. Simulation Activities

Simulation activities are another type of intervention used with medical students and residents in order to educate them about transgender patients. One such study used internal medicine residents and found that they reported a lack of confidence in psychosocial skills (e.g., rapport-building, demonstrating the use of affirming language) with transgender patients and discomfort with the biomedical aspects (i.e., hormone management and health screenings) of transgender healthcare [72]. The results of that study led to a proposal for future simulations to take a more staged approach, starting with basic psychosocial skills like the use of affirming language and then progressing to more complex skills, such as initiating and maintaining hormone replacement therapy for transgender clients, in order to address providers with different levels of skills. 

Another simulation activity included a broader range of interprofessional students (i.e., those in medicine, nursing, occupational and physical therapy, physician assistant positions, social work, and healthcare administration) and used team-based education to teach about transgender health. These were found to produce high self-reported competencies with preparedness for treating transgender patients [73]. Role-playing simulation is another type of simulation that has been employed with nursing students and was found to provide benefits in terms of comfort with advocacy and communication abilities with transgender patients and their families [74]. Simulation educational interventions are a promising tool for working to increase provider comfort and competency with transgender patients and may be useful when it is not feasible to use real patients, as they still provide a beneficial experience.

#### 4.1.5. Other Intervention Efforts

Several other methods of transgender health education interventions for providers have also been studied. One such approach used a two-hour modified jigsaw exercise that included structured cooperative learning voluntarily implemented in medical students [85]. This was found to increase knowledge and self-confidence in terms of discussing gender identity immediately post session. However, at the one-year follow-up of this work those increases were partially lost, but the knowledge and self-confidence were still higher than pre-session, which suggests at least some longer-term benefits of the intervention. This also points to a need for interventions that are longer term. 

Other efforts have been made in online training programs, including for rural primary care medical residents [82] and an hour-long online intervention for students studying healthcare-related subjects [84]. Though neither saw significant improvements in attitudes toward transgender people, the former did find that the program was sustainable and accessible for participants [82], and the latter found improvements in knowledge and perceived competence [84]. Another study gave online training on transgender health, the Transgender Health Learning Series (THLS) [88], to nursing and physician assistant students; this was offered as optional, and students received course or service credit but had alternatives [83]. The THLS consists of five hour-long courses developed in collaboration with transgender people [85]. The post-surveys of participants found improvements in knowledge and perceived competence but not in attitudes [83], similar to previous results [82,84]. 

### 4.2. Interventions Conducted on Existing Providers

Though the majority of educational interventions around transgender health have been performed with medical students and residents, there have been a few studies that have focused on more experienced, existing providers. One study gave an hour-long educational presentation on transgender healthcare to primary care providers and did see significant improvements in their knowledge, confidence providing care, and attitudes toward transgender patients [89]. Another similar brief intervention used an educational video to teach essential topics in transgender and non-binary health to both existing providers and medical students; this intervention was also found to produce improvements in knowledge and comfort in caring for transgender and non-binary patients [90]. Another study employed a more longitudinal approach, with three two-hour training sessions given to providers at an outpatient clinic over the span of four months [91]. This work found significant post-training decreases in negative attitudes toward transgender people, as well as increases in trans-related clinical skills, awareness of transphobic practices, and self-reported readiness to provide services to transgender people [91]. 

A more unique approach to continuing education was taken by one university, where they hosted a community forum on transgender healthcare that facilitated dialogue between transgender community members and HCPs (including medical school staff, faculty, and students), which was found to be beneficial for identifying priorities in transgender care in the community [92]. Engaging actual community members can be a useful way to get real input in order to work toward change. Cultural awareness and sensitivity to transgender clients’ experiences, challenges, and barriers to care are all important educational topics for providers. It is imperative that there be collaboration between clinicians, educators, and community advocates in order to achieve the best and most comprehensive care services, in terms of quality and consistency, for transgender clients [93]. Likewise, the current scarcity of research on working to improve existing HCPs’ care of transgender patients should make it a high priority for researchers in future work.

## 5. Discussion

Transgender people in the USA face many challenges as a vulnerable population. These include access to, and adequate provision of, healthcare. Their experiences in healthcare have been examined, along with barriers to healthcare accessibility. The previous educational experiences and attitudes of HCPs toward transgender people in the USA have been reviewed, in addition to interventions that have attempted to make progress in these areas. The limitations of the reviewed literature will now be discussed in order to make recommendations for future research and practice to better support transgender healthcare needs in the USA.

### 5.1. Continuing Medical Education Research

While the importance of transgender health in medical and other professional school curricula cannot be overstated, the area of continuing medical education (CME) for existing physicians should be another area for researchers to focus on. The goal of CME is to “facilitate successful performance of practitioners in diverse practice characteristic of professional work” [94]. A previous systematic review of the CME literature found that widely used methods like conferences had little impact on directly improving professional practice [95]. However, more recent reviews found small effects of CME-related activities on improving physician practice and patient outcomes [96,97,98,99]. The most reliably positive impacts of CME were on physician performance more than on patient health outcomes, but more interactive, longer-term, and multiple-exposure forms of CME have been found to lead to greater improvements in both [99].

### 5.2. Limitations of Previous Interventions

Though the aforementioned educational interventions on transgender healthcare have provided a solid foundation for future research, they are not without limitations. Notably, most interventions were short (around a single one-hour session), the effects of the interventions were only measured in the short term (usually immediately following intervention), and involved small sample sizes. Those studies that did measure longer-term post-intervention effects had mixed results, with only a few showing an enduring efficacy [78,85], while others did not see lasting improvements [68]. Future studies should strive to employ randomized, controlled trial designs in order to draw stronger causal interpretations from the interventions. The quality of reported instructional hours further limits previous work, as not all instruction is created equal, so the quantity itself does not tell the full story regarding the efficacy [58]. Another limitation is that the previous work has focused primarily on current medical students or residents, leaving a dearth of unexplored research involving more experienced HCPs. 

The effects of the interventions generally centered on improving knowledge and comfort, while less consistent improvements were seen in terms of attitudes toward transgender people. This warrants further investigation into HCPs’ attitudes due to previously reported negative experiences with HCPs by transgender people. This is further complicated by the issue of measuring attitudes toward transgender people, with the validity of some scales called into question [100]. The possible efficacy of interventions may also be limited by the existing beliefs of HCPs, as not everyone will change beliefs and behaviors to improve transgender healthcare, but education around, and exposure to, transgender clients can be a good start [56]. Moreover, as mentioned above, combatting forms of bias requires nuance, and a variety of approaches may be needed in order to do so.

### 5.3. Suggestions for Future Research and Practice

In the future, researchers should focus on several strategies to better support transgender patients through the education of both trainees and existing HCPs. The ways in which they can do this include using multi-session interventions rather than one-time learning opportunities, following up with the HCPs more than once, and over time, in order to measure the lasting efficacy of interventions [87], using a combination of different interventions (e.g., simulations, seminars, interactive discussions, lectures, etc.), seeking out and integrating input from the transgender community in their interventions, and recruiting larger sample sizes for more powerful studies. However, it should be noted that all of these strategies can be costly and time-consuming, which calls for a need for more funding to be put toward developing and implementing transgender healthcare training. Gender-affirming care has been found to be effective in improving health and mental health outcomes among transgender clients (see [101,102,103,104]), and this fact is vital for HCPs [105].

Additional strategies that could be beneficial for training HCPs working with transgender individuals include applying adult education research, such as the use of peer trainees (see [106,107,108]) and/or mentorship [109]. Previous work on cultural competency training in health professionals has also raised concerns in terms of the research quality [110], which calls for higher-quality methods in future studies. A diversity in providers has been found to be beneficial, as well (see [111]), which could be studied in a transgender health context, with patient outcomes examined for transgender-identified providers compared to non-transgender-identified providers. The two groups could be compared to determine the level of cultural competence required to provide the same level of care that transgender-identified practitioners may provide, for improved patient outcomes.

In terms of future research specifically on CME, the mechanisms of action by which CME allows for an improved physician performance and client health outcomes should be made clear through the use of more sophisticated theoretical and methodological bases [99]. This may be worked toward through the consideration of a wider range of factors that impact HCPs’ performance and patient outcomes, such as social, political, and organizational factors [99]. More focus should be placed on patient outcomes, as these have been found to show lower improvements following interventions compared to provider outcomes, which show greater improvements after cultural competency training [112]. Clinical issues involving gaps in quality care for patients should be highlighted in CME, and medical practitioners should be shown how to identify gaps in practice and methods for addressing the issues [113]. These CME programs could be supplemented using post-training deliverables (i.e., improvement plans developed based on CME), which could then be submitted as part of the recertification process to continuing education credit evaluators [113]. One such organization that could be worked with is the American College of Physicians, which has implemented a quality improvement program (see [114] for further reading).

Future studies should also be sure to incorporate scales to measure attitudes toward transgender people that have proven valid, such as the Attitudes Toward Transgender Men and Women [100]. New scales could also be developed in order to specifically measure HCPs’ attitudes toward providing gender-affirming care specifically, and not just their attitudes toward transgender people in general [83]. Previous work has relied on explicitly reported attitudes toward transgender people; future work could use implicit bias measures, namely the Implicit Association Test, which has been tested in the general population and has been found to predict beliefs about gender and transgender people [115], to specifically investigate healthcare student and provider attitudes toward transgender people. The effectiveness of interventions could also be examined through surveying the patients of the HCPs undergoing interventions; doing so would provide a stronger validation [80] compared to self-assessed knowledge and competence/attitudes. Beyond the research, the education on transgender health should be formally integrated into more medical and professional school curricula as required learning rather than optional supplements. 

## 6. Conclusions

The current paper has reviewed transgender patient experiences in the USA, as well as a variety of educational interventions conducted with HCPs that sought to improve their education and, ultimately, improve the attitudes toward, and the experiences of, transgender patients. The strategies and future directions discussed here apply to the USA context and may not necessarily be generalized. Other countries provide different contexts and should be studied in the future literature in order to provide tailored recommendations. In terms of the USA context, although there have been positive results found, there is still much room for improvement. Researchers and HCPs alike need to work to both develop and implement practices that provide more affirming and welcoming healthcare spaces for transgender people.

## Data Availability

Not applicable.
